# The Probable Causes of Malaria and Epidemical Diseases

**Published:** 1868-09

**Authors:** George W. Rains

**Affiliations:** Formerly Professor of Chemistry in West Point, U. S. A.


### Selections.

---

THE PROBABLE CAUSES OF MALARIA AND EPIDEMICAL DISEASES. *Read before the Medical Society of Agusta, Ga., July 18th,* 1866. By Col. GEORGE W. RAINS, formerly Professor of Chemistry in West Point, U. S. A.

*Mr. President, and Gentlemen of the Medical Society*:

In speaking on the subject for this afternoon's discussion, I think it proper that I should first state my views as to the nature of force, so far as to explain the term *points of force* employed in the question proposed. To give a comprehensive definition, I should say that Force is anything which can cause motion, either in bodies or in the ultimate atoms of matter. This definition would then include as forces sound, heat, magnetism, light, electricity and chemical affinity, actinic or photographic rays, nervous influence and vital action, as well as gravitation, cohesion, etc. These primary forces act in connection with the ultimate atoms of matter to which they impart motion, and as each atom is supposed to be indefinitely small, we may regard them practically as mere mathematical points. What may be the actual dimensions of such particles, is a question which has never been answered. We know, however, that if we reckon them up in weight, in parts of a grain, or in dimensions, in parts of an inch, we should have to employ the term billions, at least, or more probably quadrillions; indeed the belief is entertained by many, and is daily gaining ground, that they have no dimensions at all, but are truly mathematical points, whence emanate attractions and repulsions. The air around us is composed of such atoms, each one associated with heat, light, and electricity; hence each atom may be regarded as a mere point, from which radiate forces in straight lines, or rays in all directions; or we may say the air is composed of points radiating forces, or simply points of force.

The belief has generally been entertained that there are marked differences and broad distinctions between the inorganic and organic worlds, as well as between the vegetable and animal kingdoms. I think, however, that to-day the certainty of such conclusions is far less strong than it was considered some fifty years ago. Every day we appear to break down some of the separating barriers, until some believe that after a time there will be but slight obstructions left, if indeed there shall be any left at all. Between amorphous matter and a highly organized vegetable, there exists a deep gulf of separation; also between a tree and an animal; but between the lowest vegetable structure and the highest inorganic form the difference is not so striking; the lower organisms mingle insensibly together, so at times it is difficult if not impossible to say which is the vegetable and which the animal.

In the frosty forms on windows in freezing weather, we have beautiful representations of some of the palm species; and when nitrate of silver is crystallized and viewed in the microscope, and colored green by polarized light, it would be taken by any one not acquainted with the facts to be a growing shrub or sprig of moss, the representation being perfect. It is said that if bichromate of potash be crystallized on a film of gelatine, not only is a tree-like form produced, with its limbs and branches, but actually rhomboidal fruit appears to hang pendant in the foliage. Again, granules of starch, which is a vegetable structure, regularly formed by growth in the cells, when viewed in the microscope by reflected light, looks very much like transparent rounded crystals. The constituents of all organisms, whether vegetable or animal, are mineral elements, and it was long supposed that the peculiar combinations of those elements selected by the vegetable and animal kingdoms could not be formed in the laboratory, but were the exclusive results of life-action. It is now well established, however, that this was an error, as not less than one thousand of such organic compounds, such as urea and its compounds, acetic acid, methyle, amylene, the alcohols, napthalene, glycerine, grape sugar, etc., have been produced artificially or without the agency of vitality.

All vegetables and animals are mainly composed of air and water, the latter holding carbonic acid in solution, and only two or three per cent. of other matters. Motion can not be held as the characteristic of the organic world, since certain vegetable germs have no motion, and finely-divided matter diffused in liquids in many cases has a distinct molecular movement, and the crystals of camphor in small fragments move about on the surface of water with considerable activity, closely resembling the movements of some of the infusoria. Crystals grow in size by attracting such particles in the surrounding solution as are suitable to their formation; the same thing is done by vegetable organisms. The hard seeds of plants, when placed in a watery solution of such elements as are required, will decompose, or its constitution will be broken up, by attracting and assimilating the proper materials, and the result is a shrub or plant. Also, if a piece of zinc be placed in a solution of acetate of lead, it will partially dissolve, and at the same time will attract thè precipitated atoms of the metal in solution, and the observed result will be the growth or formation of a beautiful metallic shrub.

In making these comparisons, I do not pretend that we are unable by examination to say which is the organic form and which the inorganic structure, but merely to draw attention to the fact, that the broad distinctions hitherto held as existing between the organic and inorganic worlds no longer exist; that as the animal forms imperceptibly pass into the vegetable, so those of the latter, when our knowledge shall be more extended, may also be found to gradually fade into inorganic structures.

The power which attracts the atoms of amorphous matter together we call chemical affinity, or chemical or electrical force, and that which holds together particles of the same kind we term cohesive force; crystalic force attracts and unites the atoms into regular forms, and vital force attracts and unites the proper atoms into organized forms. It is not seen why this latter force should be considered as differing from the others, except in being of a higher order. They are all means or agents in the hands of the great overruling Intelligence to work out His designs and fulfil His plans.

Having thus set forth in detail, as far as my limits will permit, what I understand by the term force, and points of force, I will now pass on to the consideration as to whether such points of force may not exist intangible to the senses which may be capable of self-division, equivalent to propagation or multiplication.

The first appearance of a new existence or formation, whether it be animal, vegetable, or mineral, is a minute transparent point just perceptible to the highest powers of the microscope. As it grows or expands by assimilating the suitable surrounding elements, if it be a mineral, it assumes some definite crystalic form; if an organism, it is observed to be more or less globular in form, and consisting of an outer enveloping sac enclosing an albuminous liquid or semi-liquid, and having a dot or nucleus in its centre. In the earlier growth, in some instances, there appears neither enveloping sac nor nucleus, but merely a rounded mass of gelatinous substance. Such is the beginning of all life, from the simplest photophyte to the varied and exceedingly complex structure of man, as is well known to those whom I now have the honor of addressing. In the formation of the crystal the ultimate composing atoms or points of force appear to act in certain directions with more energy than in others; and hence, in their association, the resulting form is in general angular, or developed with regard to certain fixed lines, axes, or poles. In the growth of the organic structure, the vital or formative force appears to act or radiate from the centre, in the primitive vesicle. If we suppose a single point of force acting in all directions, but with power lessening as the distance increases from the centre, and that to a certain distance it is attractive to certain elements in the surrounding medium, but beyond such limit the attractive force becomes too feeble for such an effect, then there would result a globular form of organized matter, or in other words a living existence of the simplest kind.

In the further development of the simple organisms, a constriction begins to appear apparently without cause, which, becoming more and more defined, ultimately ends in dividing the cell or individual into two perfect cells or existences. In this operation, the point of force acting from the centre, and represented by the dot or nucleus, began to divide itself into two parts simultaneously with the first appearance of the constriction around the cell or globule. It would thence appear that the division of the central point of action into two parts necessarily determined the division of the enveloping cell into two individuals. Each new cell thus formed repeats the process of forming two separate cells, and thus proceeding in a geometrical ratio until, within a few hours, the number perhaps may be counted by millions.

In some cases, if two of the cells after separating touch each other, the point of meeting dissolves away gradually, until the two cells entirely mingle their contents, and a single cell is the result. The product of such fusion or conjugation is remarkable, for the compound cell dries up into a hard grain or spore, which may last indefinitely, floating about in the air, or under favorable conditions will develop cells similar to the original ones, which by binary division will form new structures of agglomerated cells covering extended areas. The growth of such plant is favored in some cases by cold and damp, whilst the union of two separate cells into one, or the act of generation resulting in the spore, is promoted by heat and dryness. Thus, during the Spring and cooler part of the Summer, such kind of vegetation may grow luxuriantly, but it requires the hotter and dryer portion of the season to develop the sporules, which, from their minuteness, are carried off by the breeze. The size of the spores in some species of the cryptogamia is very small: thus I have measured the average spore of the stysanus caput medusæ and found it about the one five-thousandth of an inch in diameter, or so small that such a drop as would adhere to the point of a needle would contain sufficient space for fifteen millions. Indeed, there appears to be no limit to the smallness of size in the minutest organisms of infusorial life, and as the power of the microscope is increased it brings new, and before invisible, points of life into view, just as the increased powers of the modern telescope resolves into mathematical shining points the distant nebulæ of space. It is then clear that organisms capable of self-division and propagation may and probably do exist beyond the utmost powers of our best microscopes, and hence that countless sporules *may* float in the air invisible to man, although assisted by the highest powers of modern art. Such existences we may call sporules, atoms, or points of force, and they may ever remain as invisible as the particles constituting the various odors, and can only be known to exist, like the latter, by their effects on the exceedingly subtle nervous or vital forces.

I will now examine into the effects on the nervous and vital forces produced by the introduction of sporules into the circulating system, either by inoculation or by being drawn into the blood by inhalation of the air in which they float. It will be premised that the vital force of an organism attracts each atom of its structure, or that each atom is enveloped by the vital force precisely as it is enveloped or conjoined to the force of heat, and when such atom shall be removed from the general system, it carries with it its corresponding envelopes of associated forces; hence the loss of a certain number of atoms composing the structure of a living organism would result necessarily in the subtraction of an equivalent amount of vitality.

On the introduction of the sporule or germ into the blood, a contest immediately arises between the vital forces of the spore and those of the blood: the former endeavoring to attract to itself the nutritious elements of the latter suitable to its development. If the animal be in vigorous life, all the forces of its system are in full activity, and the spore seeks in vain to overcome the resistance opposing the disintegration of its constituents. If, however, the number of sporules or germinal cells introduced into the blood be so great that their combined power exceeds the resisting forces of the latter, or if from other cause the vital force of the blood should have been previously reduced too low, then the attractive power of the sporule will succeed in drawing to itself such of the elements of the blood as it may require, and the development of cells having commenced, proceeds with accelerated rapidity by self-division and reproduction.

Thus the constitution of the blood is gradually disorganized: each removed atom has taken with it a portion of the vital force; the energies of the system become impaired; the portions of the blood disorganized by the attractive forces of the sporules, withdrawing some of its constituent elements, remain as foreign matter which must be eleminated. Thus additional work is thrown on the excreting organs, already weakened by the abstraction of a portion of the vital force from the general system. The loss of vital energy, and the deteriorated condition of the nutrifying fluid, prevents its full normal action, and an increased waste of tissues takes place, the components of which have also to be removed from the system by excretion or combustion. This disturbed condition of the general function of the system results in fever, a particular discussion of which, as well as of the chill which precedes it, does not immediately belong to the subject under consideration.

What is that which vitiates the air in particular localities, producing intermittent, and bilious affections? has been a question probably from the earliest ages of civilization. The bad air or malaria of the Pontine marshes ages ago caused the wealth and fashion of Rome to leave the pestilential atmosphere of the city during the two warm months of Summer, and seek a purer breathing medium in healthier localities.

Everywhere, when the warmth, moisture and fertility of the soil are favorable for the rapid production and decay of vegetable and animal life and tissues, we find malaria. Thus marshes, swamps, and stagnant water in warm climates, or during the warm seasons of the year, infect the surrounding air, as is well known; even in healthy localities, if the firm soil for the first time is broken up, thereby exposing innumerable vegetable fibres to rapid decay, and perhaps also releasing imprisoned sporules and germinating points, malarial influence is experienced. Thus in the comparatively healthy districts of South Florida, in 1848 and 1849, I found in every case where the soldiers broke up the ground, whether for the purpose of policing and ditching the camps, throwing up earth-works, or in gardening, malarial fevers ensued, notwithstanding every precaution was taken to preserve health; and when, at certain points considered very unfavorably situated, I took the precaution to prevent any disturbance of the soil, the men under my command enjoyed good health for such localities. Thus new agricultural countries must abound in malaria, because of the breaking up of new soil, and old cultivated districts become healthier, because of the gradual decomposition of those organic matters which were peculiarly favorable for the generation of poisonous fungi, sporules, or exhalations. Hence even in old districts the dwelling-houses should not be in the midst of cultivated grounds, but as far as practical removed from them; not the *sides* of hills or elevations fronting on plantations, swamps, marshes, creeks, or rivers, but beyond the crests of such rising grounds. The warm rays of the sun striking such elevated places with more freedom than the shady bottoms, cause a rarefaction of the air, which rising more or less vertically, has its place supplied by the poisoned air of the low grounds. Thus, a dwelling on the side of a hill, unless protected by a thick growth of trees, would be exposed to a daily current of malarial atmosphere from the bottom lands, and would be in a worse situation than if located on the lowest adjacent land.

Warmth, moisture, and decaying vegetable matter are suitable conditions for the growth, in general, of cryptogamia with the rapid evolution of the innumerable sporules of the fungi. From the circumstances attending the production of malaria, it would appear to be either air containing floating sporules, germinal vegetable or animal cells, points of force capable of propagation, or air mixed up with the diffused gases arising from organic decomposition.

At Mont-faucon in Paris, there are extensive enclosures called Huacker-yards, where thousands of animals are slaughtered as worthless, or the dead bodies carried there to save the skins and to allow the carcasses to putrefy for the purpose of manure. From this mass of putrescent animal substances are evolved all the gases given off by vegetable decomposition, and several others in addition, of the most nauseous and disagreeable odors, involving ammonia and the compounds of sulphur and phosphorus. Notwithstanding, the numerous workmen with their families who live in the midst of this most offensive effluvia preserve excellent health; indeed, the family of one of them named Friand, consisting of his wife and five children, were in remarkably robust health, although they had all the year round worked and slept in a place which was actually unapproachable, on account of the stench, to the members of the Commission appointed by the Government of France to examine into the matter. Moreover, these workmen live to be old men and women, many above seventy and eighty years of age.

Again, all the gases given off by vegetable decomposition are breathed at times in the laboratory of the chemist, in a more concentrated condition than ever takes place in the natural decomposition of such bodies, without any malarial effects whatever. It is true, the long breathing of carbonic acid and carbonic oxide, when in appreciable amounts, will lower the vitality of the system, and perhaps, in conjunction with other causes, may at times produce typhoidal disease, like anything else which would diminish the energies of the body whilst certain conditions prevailed; but I presume there exists no accredited case of a healthy person living in a healthy atmosphere who has had malarial fever from the effects of any single or compound gas.

In the matter of epidemical poison in the air, there is still less foundation to suppose it results from the admixture of a deleterious gas or exhalation. It is well known that Asiatic cholera was engendered in the Souderbund marshes of the Ganges in India, and thence spread through the English army in 1817, whence for the first time it became the terror of the civilized world. In the same district of country a pestilence like the plague, preceded by cholera, arose in 1860, and for the three succeeding years swept off many thousands of the inhabitants. Dr. Elliot, of the army, who was sent to report upon the disease, attributed it to malaria and water filled with decaying organisms.

It is evident that, whatever may constitute epidemical poison, it can not be gases, since it propagates itself, and extends from place to place, far beyond the original locus. It must be, then, either a peculiar propagating organism, propagating points of force, or an electrical phenomenon.

But how can electricity act? It is a primary force like light and heat, and like them is diffused in matter and throughout space. It can indeed energize the oxygen of the air into ozone, and can diminish its normal activity into autozone, and such changes might doubtless cause variations of health throughout wide districts of country. But why should electrical action follow particular streets of a city, or one side of a street, or one bank of a river, or follow exclusively the lines of travel? Electric force can not be carried about in the clothes, or propagate itself from house to house, or adhere to the stools of cholera patients, which is said to be a main source of its propagation. It is well known that electricity can be produced by friction of the clothes, or, indeed, by any species of molecular disturbance, but who ever heard of such producing cholera, or any other disease? The important fact that the excrements of cholera subjects are the principle sources of contagion, bears strongly on the probability of the poison being organic points capable of propagation. It may be asked, why could there not be minute poisonous atoms floating in the air or deleterious unknown gases, which would thus poison the blood and cause disease? Doubtless such may be the case, but we must admit in epidemics such points or deleterious gases to be capable of propagation, otherwise the disease produced would soon terminate for want of cause, and if propagative, then they come under the definition either of organisms or propagative points of force.

There are three ways of poisoning the blood, resulting in disease or death, viz: First, by fermentation caused by the inoculation or reception of bodies capable of producing such action; Second, by chemical combination with the foreign substance; and Third, by what is termed catalytic action. Fermentation is the breaking up of the constitution of the blood by organisms which feed upon some of its elements, thus separating into new compounds such portions as are acted upon; the ultimate of fermentation is putrefaction and dissolution. Some of the ferments are vegetable, and some animal; generally the decomposition of animal tissues is effected by infusorial animalcules, the smallest commencing the operation. viz.: the monas crepusculum, and bacterium termo, according to the researches of M. Pasteur, a distinguished French chemist. The size of these animated points is so minute that the smallest specimens imperceptibly fade away under the highest powers of the microscope, and are only known to exist at certain points by the slight movement of the liquid which surround them. Thus I have at first, in the sanguinious matter from an ulcer, seen only a kind of motion in the semi-liquid mass with a magnifying power of four hundred diameters, but with a careful increase of power and arrangement of light, I have detected countless thousands all in active movement. An idea has been attempted to be given of the relative size of these excessively minute organisms, by saying that thousands of them might sail side by side through the eye of an ordinary needle.

The poisoning of the blood by chemical combination with a foreign substance is well seen in cases of poisoning by arsenic or corrosive sublimate, which unite with the albuminous tissues or parts, and thus, rendering them solid, incapacitate for further vital operations. This action of these two substances renders them virulently poisonous to all life, for every organized body contains albuminous compounds essential to its structure. Thus, arsenic, or chloride of mercury, in solution, when given in small doses, would completely destroy the malarial spores or germinating cells, which may have been absorbed in the blood. The blood globules being larger, and hence supposed to have a larger amount of vital resistance, would not be sensibly affected unless the amount taken should be too large, in which case they also would be killed.

What is called catalytic action is the production of a certain effect in compound bodies, without any apparent change in the agent employed, and is probably an electrical phenomenon. Thus a mixture of oxygen and hydrogen gases may remain perhaps indefinitely without change, but on the introduction of a clean piece of platinum foil they immediately combine and form water. If a piece of metal be in the slightest degree electrified, it will cause the explosion of fulminate of silver. If the blood be inoculated by an organic poison, as that of a snake, an immediate action commences, and in a short time the vital force, as such, is destroyed. This species of poisoning is entirely distinct from that of fermentation or malaria, for whilst the latter find their proper field of action in a debilitated, nervous condition of the system, and consequent weak resisting power, the former acts with greatest energy when the nervous force is in the best condition. Thus it is said to take less organic poison to destroy life in a vigorous organization than in a feeble one. In other words, the poison acts directly on the nervous and vital forces, and its action is in proportion to their energy.

This would seem to indicate a possibility that those forces could be so lowered in polarity, by being brought in contact with certain agents, as to change their nature or become transformed into others, just as light, heat, and electricity are correlative, or capable of being converted the one into the other. The fact exists that the nervous and vital forces gradually disappear under the action of such poisons, and as it is held that no force can be lost under any circumstances, the question arises, what has become of them.

Alcohol, and the alkaloids, such as quinine, morphine, nicotine, etc., also appear to act directly on the nervous force; their first effects being stimulative, or increasing the energy of the nervous power, they would hence appear as the antidotes of the above kind of poisons. There must be floating in the air, in the neighborhood of localities favorable for their propagation, a number, more or less great, of minute animal organisms, the dead remains of which must be absorbed in the lungs, and there act in some measure like particles of putrid blood, poisoning the system. The same conditions, favorable for the development of vegetable organisms, would favor the production of such animalculæ, and hence attending malarial poison of the blood would be the organic poison affecting directly the nervous force. Hence, if arsenic, mercury, etc., be used to destroy the vegetable germs, then alcohol, quinine, morphine, etc., would be indicated as the proper agents to neutralize the action of the decaying animal germs; or, as the two kinds of poison probably prevail together, then a mixture of the two species of antidotes would suggest itself for malarial influences, such as doses of arsenic and brandy, or arsenic and quinine.

Arsenic, mercurial compounds, and the alkaloids, have long been employed as the proper remedies for malarial fevers, and it is interesting to trace out the chemistry of their use.

As regards the supposition that malaria and epidemics may be caused by organisms floating in the air, I will here quote the substance of the remarks of Dr. G. Robinson, in an address made to the British Association, 1863. The author alluded to the circumstance of the analogy between many of the phenomena of fevers and other zymotic diseases, and the ordinary process of fermentation having been perceived and recognized by Hippocrates and the oldest writers on medicine. Their idea was, that a poisonous ferment existing in the atmosphere, entered the mass of the blood and induced in it a series of changes, which gave rise to the excessive heat and other peculiarities of that class of disease. At the present time, this doctrine, modified by the discoveries of Liebig, and other chemists, has been adopted by most physicians, and forms the basis of the classification of disease framed by Dr. Farr, and used by the registrar-general. It thus supposes living germs to exist in the atmosphere, which, when introduced into the body, give rise to a specific and regular series of morbid actions pursuing a definite course in a definite time, as in small pox, those germs being disclosed and multiplied, and producing others capable of reproducing in other bodies the same succession of changes (An. Sci. Dis., 1864.)

I trust the foregoing remarks, however inadequate in elucidation, will prove suggestive, and assist in drawing attention to a subject of great interest to mankind.

